# Children of parents with different severities of mental health conditions have higher risk of somatic morbidity: a Danish nationwide register-based cohort study

**DOI:** 10.1186/s12889-023-15714-8

**Published:** 2023-05-03

**Authors:** Camilla Klinge Renneberg, René Børge Korsgaard Brund, Signe Heuckendorff, Bodil Hammer Bech, Kirsten Fonager

**Affiliations:** 1 Department of Social Medicine, Aalborg University Hospital, Havrevangen 1, 9000 Aalborg, Denmark; 2 Psychiatry Region North Jutland, Aalborg, Denmark; 3 Department of Public Health, Aarhus University, Aarhus, Denmark; 4 Department of Clinical Medicine, Aalborg University, Aalborg, Denmark

**Keywords:** Parental mental health, Mental health, Maternal mental health, Paternal mental health, Child health, Somatic morbidity, Mortality, Parenting, Inequality

## Abstract

**Background:**

Children with the most severe parental mental health conditions have an elevated risk of numerous adversities including somatic morbidity. However, there is no knowledge concerning physical health in most children affected by parental mental health conditions. Therefore, the aim was to examine the association between different severities of parental mental health conditions and somatic morbidity in children of different age-groups and further explore the combinations of maternal and paternal mental health conditions on child somatic morbidity.

**Methods:**

In this register-based cohort study, we included all children born in Denmark between 2000–2016 and linked parents. Parental mental health conditions were categorised into four severity groups (no, minor, moderate, and severe). Somatic morbidity in offspring was categorised into broad disease categories corresponding to the International Classification of Diseases. We estimated the risk ratio (RR) using Poisson regression, of the first registered diagnosis in different age-groups.

**Results:**

Of the around 1 million children in the study > 14.5% were exposed to minor parental mental health conditions and < 2.3% were exposed to severe parental mental health conditions. Overall, the analyses revealed a higher risk of morbidity in exposed children across all disease categories. The strongest association was observed for digestive diseases in children aged < 1 year exposed to severe parental mental health conditions (RR: 1.87 (95% CI: 1.74–2.00). Generally, the risk of somatic morbidity increased the more severe the parental mental health conditions. Both paternal and especially maternal mental health conditions were associated with a higher risk of somatic morbidity. The associations were strongest if both parents had a mental health condition.

**Conclusion:**

Children with different severities of parental mental health conditions experience a higher risk of somatic morbidity. Although children with severe parental mental health conditions had the highest risk, children with minor parental mental health conditions should not be neglected as more children are exposed. Children with both parents having a mental health condition were the most vulnerable to somatic morbidity and maternal mental health conditions were more strongly associated with somatic morbidity than paternal. More support and awareness of families with parental mental health conditions is highly needed.

**Supplementary Information:**

The online version contains supplementary material available at 10.1186/s12889-023-15714-8.

## Introduction

Two in five children grow up with parents suffering from mental health conditions [1] which adversely affects the children due to multiple deprivations [2]. However, only recently improving these children’s lives and well-being has become a public health priority [3–5]. Children’s physical health is highly important as illness and school absence entail detrimental consequences on the child’s social and academic development as well as a strain on the entire family. Furthermore, health inequality in childhood often perseveres into adulthood [6, 7]. Still, most information obtainable regarding children exposed to parental mental health conditions concerns their higher risk of developing mental health conditions themselves [5, 8, 9]. Notably little is known about these children’s physical health, even though unfavourable experiences in foetal life and childhood, such as exposure to parental mental health conditions, can cause a stress response, which is suggested leading to immune dysregulation [10–12]. Furthermore, mental health conditions might also have an impact on parenting behaviour [13–16] which, like environmental adversities, could be a component in children’s physical health [17, 18].

Studies examining somatic morbidity in children exposed to parental mental health conditions have mainly focused on mothers with depression, in particularly postpartum depression, and are mostly limited to asthma, injuries, and obesity [5]. Few other studies also indicate higher risk of epilepsy [19] and eczema [20, 21] in children of mothers with depression or schizophrenia. Thus in general, the father seems vastly neglected in the literature despite an increasing emphasis on paternal mental health conditions adversely affecting development upon offspring [22, 23].

To our knowledge, merely two studies have examined the risk of somatic morbidity across several disease categories in the offspring of parents with mental health conditions. Both studies found a higher risk in most of the categories for the examined age-groups (0–6 years [24] and 0–30 years [25]). However, these studies only focused on severe parental mental health conditions such as schizophrenia and bipolar disorder [24, 25]. Since only very few (2.2%) of the children with parental mental health conditions in Denmark have parents with a severe mental health condition [1], there is no knowledge concerning physical health in the majority of children with parental mental health conditions.

### Aim

In order to accommodate the lacking knowledge of physical health in children affected by parental mental health conditions, this explorative study aimed to describe the morbidity in different age-groups of children with parental mental health conditions. Further, we aimed to examine whether there were differences in the risk regarding the severity of parental mental health conditions and explore the combinations of paternal and maternal mental health conditions concerning children’s somatic morbidity.

## Methods

### Design

**Fig. 1:**
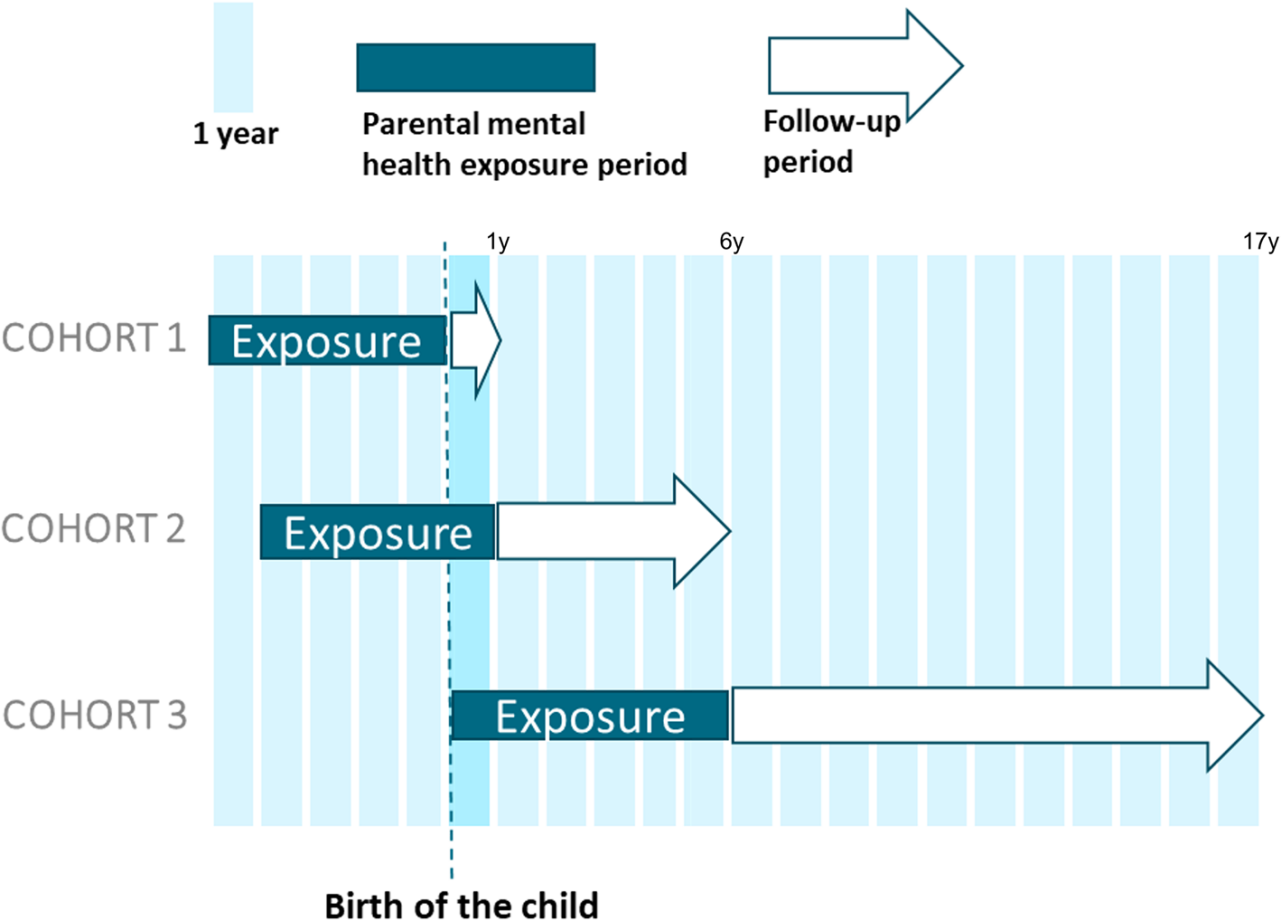
Illustration of the study design with three embedded cohorts. Legend: Children were followed in each cohort from baseline (start of the arrow) until end of the arrow or until their first registered diagnosis in each disease category, death, emigration, or end of follow-up on 31 December 2017, whichever came first

This study was designed as a Danish nationwide register-based cohort study, with three different embedded cohorts. These cohorts were identified as cohort 1, encompassing the infant year (i.e., children below the age of 1 year), cohort 2 covering the preschool years (i.e., children between the ages of 1 to 5 years), and cohort 3 representing the schoolyears (i.e., children between the ages of 6 to 16 years). See Fig. 1.

### Setting

Danish health care is universal and tax funded. In primary care, the general practitioner serves as a gatekeeper to secondary (specialised) care including referrals to in- and outpatient hospital care [26].

### Data sources

Information on children’s somatic morbidity was obtained from the Danish National Patient Registry [27]. Data on the parents’ mental health condition was obtained from the following Danish nationwide registers: From the Danish National Patient Registry [27] we collected information on psychiatric disorders diagnosed at any Danish psychiatric hospital. The Danish National Health Service Register [28] provided information on contacts to general practitioners, private psychiatrists, and psychologists. From The Danish National Prescription Registry [29] we obtained information on redeemed prescriptions at Danish Pharmacies.

Data regarding the birth of the child, death, emigration, and residence of both the parents and the child were obtained from Statistics Denmark’s register on the population.

Statistics Denmark conducted the anonymisation of data and linkage between databases on an individual level using the personal registration number, a unique identifier assigned all residents and used in all Danish public registers [30].

### Study population

We identified all children born in Denmark in the period between 1 January 2000 to 31 December 2016 and their corresponding parents. Regarding the three embedded cohorts: cohort 1 included all liveborn children in Denmark in the period. Children were included in cohort 2 on their 1^st^ birthday and included in cohort 3 on their 6^th^ birthday (see Fig. 1). Thus, children could appear in more than one cohort.

Children were excluded if their parents could not be ascertained for, in the specific exposure period or if the children were not living in Denmark at baseline for each cohort. Furthermore, we planned to exclude children if both parents had missing personal registration number. However, all mothers could be linked.

### Variables

#### Exposure

Mental health conditions of the parents were classified into four mutually exclusive severity groups (no, minor, moderate, and severe) as described in Table 1. The most severe mental health condition of either parent defined the exposure group of the child. For the analysis regarding combinations of paternal and maternal mental health conditions moderate and severe mental health conditions were combined, due to numerous combinations and few children exposed to severe mental health conditions resulting in a moderate/severe exposure group.

**Table 1:** Criteria for the four mutually exclusive severity groups of parental mental health conditions

**Exposure of mental health condition**s	**Definition** (measured in three different periods according to the three different cohorts) *
Minor mental health conditions	**At least one of the criteria fulfilled:** **Medication**^a^- *At least two redeemed prescriptions of:* --Anxiolytic medication (ATC: N03AE, N05BA, N05CD, N05CF) --Antidepressant medication (ATC: N06AB, N06AX) **Services at general practitioners** (GP)^**b**^ --At least two sessions of ‘talk therapy’ --At least two psychometric tests **Other services**^**b**^ --At least one contact to a private psychologist **Additional criteria** *(all fulfilled)* --No contacts or diagnoses from psychiatric hospital --No records of contact to a private psychiatrist
Moderate mental health conditions	**At least one of the criteria fulfilled:** **Psychiatric hospital**^**c**^ --Psychiatric diagnosis (ICD-10: Mental and behavioural disorders F00-99) registered at psychiatric hospital (both in- and outpatient contacts)—excluding the diagnoses defining the following severity group of severe mental health conditions **Other services**^**b**^ --At least one contact to a private psychiatrist
Severe mental health conditions	**At least one of the criteria fulfilled:** **Psychiatric hospital**^**c**^ --Both in- and outpatient contact with a registered diagnosis of either schizophrenia (ICD-10: F20-22) or bipolar disease (ICD-10: F30-31) --Inpatient contact with a registered diagnosis of either unipolar depression (ICD-10: F32-34) or emotionally unstable personality disorder (ICD-10: F60.3)
No mental health conditions	**None of the above**

^a^ Data on medication was obtained from The Danish National Prescription Registry

^b^ Data from GP, private psychologist and psychiatrist was obtained from The Danish National Health Service Register

^c^ Psychiatric diagnosis registered at the psychiatric hospital was obtained from The Danish National Patient Register

^*^Cohort 1: Exposure was measured in a period of five years before the birth of the child

^*^Cohort 2: Exposure was measured over a period of five years before the child’s first birthday

^*^Cohort 3: Exposure was measured from the birth of the child until the sixth birthday of that child

Since mental health conditions often are recurrent or enduring [31], and 75% of parents in a Danish survey reported that their mental health condition lasted more than five years [32], all three severity groups in cohorts 1 and 2 were considered for a five-year period prior to baseline. To include the child’s entire lifetime until baseline, exposure of the parents was, in cohort 3, measured in a six-year period (see Fig. 1 and Table 1).

#### Outcome

Morbidity in children was categorised into eighteen overall disease categories corresponding to the International Classification of Diseases, Tenth Revision (ICD-10) [33]. Each disease category was analysed as a separate outcome within the different cohorts. Furthermore, we included death as an outcome of interest.

For the analysis regarding the combinations of maternal and paternal mental health conditions, the two most common paediatric disease categories were chosen (infectious and respiratory diseases).

Titles of the overall disease category with corresponding ICD-10 codes and short descriptions applied in Figs. 3 and 4 are available in table s1 in Supplementary. (see Additional file 1).

### Follow-up

Children were followed in each cohort from baseline until their first registered diagnosis in each disease category, death, emigration, or end of follow-up on 31 December 2017, whichever came first. Additionally, children were followed differently in the three cohorts: from birth until the day before their first birthday (cohort 1), the day on their first birthday until the day before their sixth birthday (cohort 2), and the day on their sixth birthday until the day before their seventeenth birthday (cohort 3) (see Fig. 1).

### Statistics

Within each of the three cohorts, the proportion of children with a registered diagnosis was calculated in each disease category for all severities of parental mental health conditions – that is, a father and/or a mother with either no, minor, moderate, or severe mental health conditions. The total number and proportion of children diagnosed in each category can be seen in Supplementary table s2.

Poisson regression was used to estimate the risk ratio (RR) and 95% confidence interval (CI). Some children in cohorts 2 and 3 were by design lacking complete follow-up. These children were considered without somatic morbidity if no diagnosis was registered during their actual follow-up period. Children with no parental mental health conditions were considered the reference group for all analyses.

For the analysis regarding the combinations of paternal and maternal mental health conditions on child physical health, eight different exposure groups were generated. The eight exposure groups then consisted of the different combinations of paternal and maternal mental health conditions – as for instance both the mother and the father having minor mental health conditions, or only one parent having minor mental health condition, or the mother with moderate/severe and father with minor mental health conditions and so forth.

With the aim of describing morbidity that might be overrepresented in children exposed to different severities of parental mental health conditions, no adjustments were conducted, except for the adjustment of the child’s birthyear, since the study sample extends numerous calendar years and the occurrence of parental mental health conditions increased during the study period. Each birthyear was modelled as its own baseline risk.

## Results

**Fig. 2:**
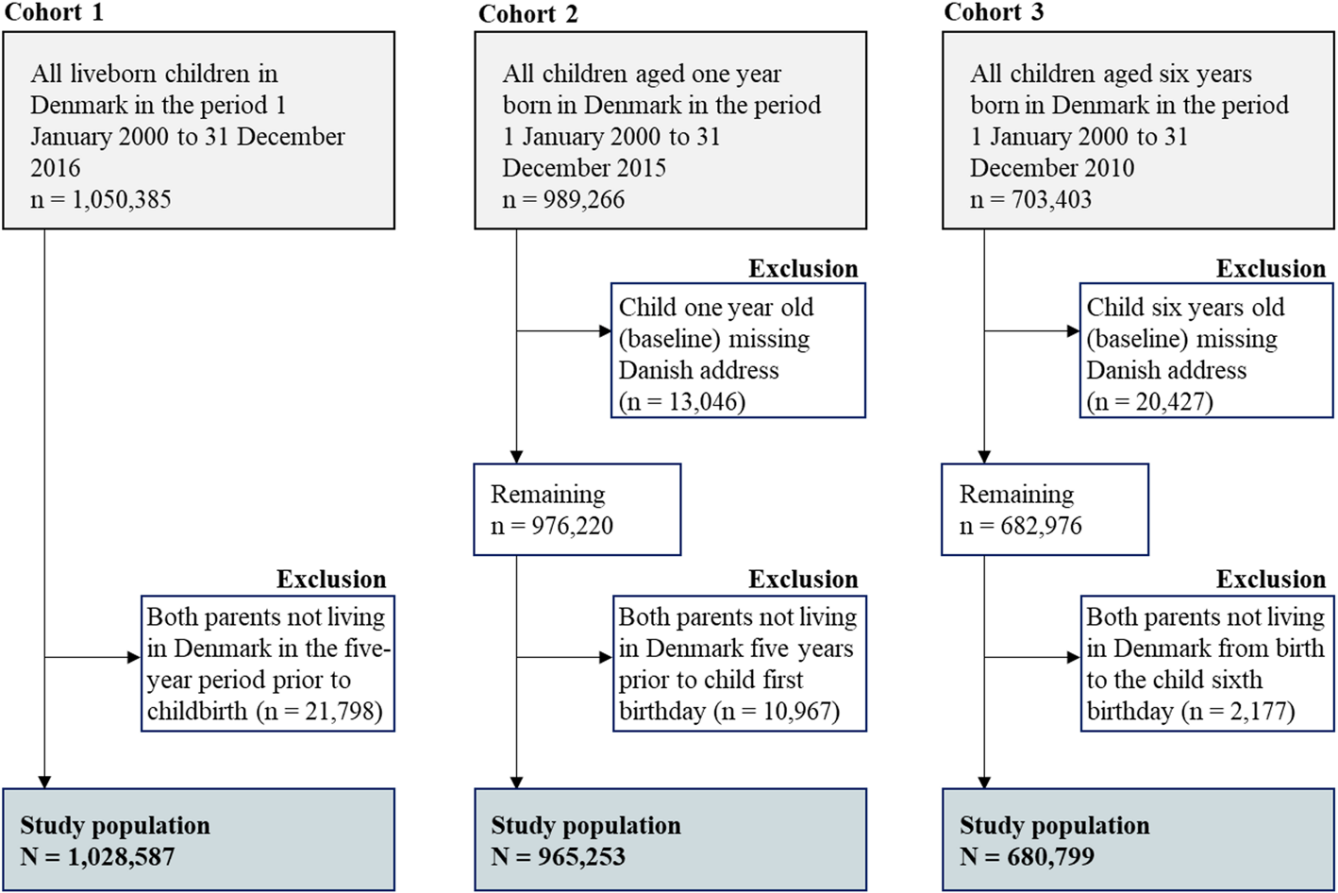
Flowchart of the inclusion, exclusion, and final study population in all three cohorts

A total of 1,028,587 children in cohort 1, 965,253 children in cohort 2 and 680,799 children in cohort 3 fulfilled the inclusion criteria (see Fig. 2).

Exposed children were especially exposed to minor parental mental health conditions with 14.5%, 15% and 22.6% in cohorts 1,2 and 3, respectively. About 10%-13% were exposed to moderate parental mental health conditions and less than 2.3% to severe parental mental health conditions.

Table 2 presents the proportion of children diagnosed in the eighteen overall disease categories along with death in the different exposure groups of parental mental health conditions. In cohort 1 (children < 1 year) the most common diagnosis originated in the perinatal period (ICD-10: P00-P99). Injuries (ICD-10: S00-T98) were numerous in cohort 2 (children aged 1–5 years) and cohort 3 (children aged 6–16 years). Furthermore, abnormal symptoms not elsewhere classified (ICD-10: R00-R99) were also highly present in children of all three cohorts.

**Table 2:** Proportion of children with a diagnosis according to parental mental health conditions

Disease grouping according to the ICD-10 classification and mortality/death	COHORT											
	**COHORT 1** < 1 year age group (*N* = 1,028,587)				**COHORT 2** 1–5 years age group (*N* = 965,253)				**COHORT 3** 6–16 years age group (*N* = 680,799)			
	**MENTAL HEALTH CONDTION**											
	**No** (*n* = 754,444)	**Minor** (*n* = 149,613)	**Moderate** (*n* = 110,795)	**Severe** (*n* = 13,735)	**No** (*n* = 703,643)	**Minor** (*n* = 144,889)	**Moderate** (*n* = 103,422)	**Severe** (*n* = 13,299)	**No** (*n* = 421,511)	**Minor** (*n* = 153,755)	**Moderate** (*n* = 90,198)	**Severe** (*n* = 15,335)
	**% (95% CI)**	**% (95% CI)**	**% (95% CI)**	**% (95% CI)**	**% (95% CI)**	**% (95% CI)**	**% (95% CI)**	**% (95% CI)**	**% (95% CI)**	**% (95% CI)**	**% (95% CI)**	**% (95% CI)**
Infectious	5.6 (5.6–5.7)	7.5 (7.4–7.7)	8.6 (8.5–8.8)	9.2 (8.7–9.6)	9.3 (9.3–9.4)	12.0 (11.9–12.2)	13.7 (13.5–13.9)	14.4 (13.8–15.0)	3.6 (3.5–3.6)	4.0 (3.9–4.1)	4.8 (4.7–5.0)	4.5 (4.2–4.8)
Neoplasms	0.5 (0.5–0.5)	0.6 (0.6–0.7)	0.6 (0.6–0.7)	0.6 (0.4–0.7)	0.7 (0.7–0.7)	0.8 (0.7–0.8)	0.8 (0.7–0.8)	0.8 (0.7–1.0)	1.0 (1.0–1.1)	1.0 (1.0–1.1)	1.0 (0.9–1.0)	1.0 (0.8–1.1)
Blood	0.2 (0.2–0.2)	0.3 (0.3–0.3)	0.3 (0.3–0.4)	0.3 (0.2–0.4)	0.7 (0.7–0.8)	0.9 (0.8–0.9)	1.0 (0.9–1.1)	0.8 (0.6–0.9)	0.6 (0.6–0.6)	0.6 (0.6–0.7)	0.7 (0.7–0.8)	0.6 (0.5–0.7)
Endocrine	1.1 (1.1–1.1)	1.6 (1.5–1.6)	1.7 (1.6–1.7)	1.9 (1.7–2.1)	2.4 (2.3–2.4)	3.3 (3.2–3.4)	3.6 (3.5–3.7)	3.9 (3.5–4.2)	3.0 (3.0–3.1)	3.5 (3.4–3.6)	4.1 (4.0–4.2)	4.1 (3.8–4.4)
Mental	0.3 (0.2–0.3)	0.5 (0.4–0.5)	0.6 (0.5–0.6)	0.7 (0.6–0.9)	0.8 (0.8–0.8)	1.2 (1.1–1.2)	1.6 (1.5–1.7)	2.0 (1.7–2.2)	0.8 (0.8–0.8)	1.3 (1.3–1.4)	1.8 (1.8–1.9)	2.0 (1.8–2.3)
Nervous system	0.6 (0.5–0.6)	0.7 (0.7–0.7)	0.8 (0.8–0.9)	1.0 (0.8–1.2)	1.3 (1.3–1.4)	1.8 (1.7–1.8)	2.0 (1.9–2.0)	2.4 (2.2–2.7)	2.1 (2.0–2.1)	2.5 (2.4–2.6)	2.6 (2.5–2.7)	2.5 (2.3–2.8)
Eye	0.8 (0.8–0.9)	1.1 (1.0–1.1)	1.3 (1.2–1.4)	1.5 (1.3–1.8)	2.4 (2.3–2.4)	2.8 (2.7–2.9)	3.2 (3.1–3.3)	3.5 (3.2–3.8)	2.5 (2.4–2.5)	2.8 (2.7–2.9)	3.1 (3.0–3.2)	3.0 (2.8–3.3)
Ear	1.8 (1.8–1.9)	2.1 (2.1–2.2)	2.6 (2.5–2.7)	2.7 (2.4–3.0)	5.2 (5.1–5.2)	6.5 (6.4–6.6)	7.4 (7.2–7.5)	7.3 (6.9–7.8)	2.6 (2.5–2.6)	2.8 (2.7–2.9)	3.0 (2.9–3.1)	2.8 (2.5–3.1)
Circulatory	0.3 (0.3–0.3)	0.3 (0.3–0.4)	0.4 (0.4–0.4)	0.4 (0.3–0.5)	0.4 (0.4–0.5)	0.5 (0.5–0.5)	0.6 (0.5–0.6)	0.5 (0.4–0.6)	0.7 (0.7–0.7)	0.8 (0.7–0.8)	0.8 (0.8–0.9)	0.9 (0.7–1.0)
Respiratory	8.4 (8.4–8.5)	10.5 (10.3–10.6)	12.3 (12.1–12.5)	13.2 (12.6–13.8)	16.6 (16.5–16.7)	20.3 (20.1–20.5)	22.7 (22.5–23.0)	23.3 (22.6–24.0)	24.7 (24.6–24.8)	28.9 (28.7–29.2)	32.8 (32.5–33.1)	32.5 (31.7–33.2)
Digestive	2.7 (2.7–2.8)	4.2 (4.1–4.3)	4.8 (4.7–5.0)	5.7 (5.3–6.1)	4.7 (4.7–4.8)	6.0 (5.9–6.2)	6.6 (6.5–6.8)	6.9 (6.5–7.4)	5.7 (5.7–5.8)	6.3 (6.2–6.4)	7.2 (7.0–7.3)	7.2 (6.8–7.6)
Skin	1.3 (1.2–1.3)	1.5 (1.4–1.5)	1.7 (1.6–1.8)	1.8 (1.6–2.1)	3.5 (3.5–3.6)	4.0 (3.9–4.1)	4.7 (4.6–4.8)	4.7 (4.3–5.1)	3.0 (2.9–3.0)	3.2 (3.1–3.3)	3.7 (3.6–3.8)	3.5 (3.2–3.8)
Musculoskeletal	0.8 (0.7–0.8)	0.9 (0.9–0.9)	1.0 (0.9–1.0)	1.0 (0.8–1.1)	3.7 (3.6–3.7)	4.2 (4.1–4.3)	4.4 (4.3–4.5)	4.4 (4.0–4.7)	6.9 (6.8–7.0)	7.3 (7.2–7.4)	7.6 (7.4–7.8)	7.2 (6.8–7.6)
Genitourinary	1.0 (0.9–1.0)	1.3 (1.2–1.3)	1.3 (1.2–1.3)	1.2 (1.0–1.4)	2.9 (2.8–2.9)	3.4 (3.4–3.5)	3.6 (3.5–3.7)	3.5 (3.2–3.9)	3.7 (3.7–3.8)	4.0 (3.9–4.1)	4.6 (4.5–4.8)	4.3 (4.0–4.7)
Perinatal	25.7 (25.6–25.8)	30.4 (30.1–30.6)	31.6 (31.4–31.9)	34.4 (33.6–35.2)	-	-	-	-	-	-	-	-
Malformations	5.6 (5.6–5.7)	6.7 (6.5–6.8)	6.9 (6.8–7.1)	7.1 (6.7–7.5)	4.8 (4.8–4.9)	5.5 (5.4–5.6)	5.8 (5.7–6.0)	6.6 (6.1–7.0)	3.4 (3.4–3.5)	3.9 (3.8–4.0)	4.0 (3.8–4.1)	4.1 (3.8–4.4)
Other symptoms	7.3 (7.3–7.4)	9.9 (9.7–10.0)	11.3 (11.1–11.5)	12.4 (11.8–12.9)	13.4 (13.3–13.5)	16.4 (16.2–16.6)	18.3 (18.0–18.5)	19.8 (19.1–20.5)	13.0 (12.9–13.1)	14.7 (14.5–14.9)	16.7 (16.5–17.0)	16.6 (16.0–17.2)
Injury	3.8 (3.7–3.8)	4.3 (4.2–4.4)	5.5 (5.3–5.6)	5.5 (5.1–5.9)	41.0 (40.9–41.1)	41.8 (41.6–42.1)	45.8 (45.5–46.1)	45.6 (44.8–46.4)	47.7 (47.6–47.9)	47.5 (47.3–47.8)	50.0 (49.7–50.3)	49.0 (48.2–49.8)
Mortality/Death	0.4 (0.3–0.4)	0.4 (0.4–0.5)	0.5 (0.4–0.5)	0.5 (0.4–0.6)	0.1 (0.1–0.1)	0.1 (0.1–0.1)	0.1 (0.1–0.1)	0.1 (0.1–0.2)	0.1 (0.0–0.1)	0.1 (0.0–0.1)	0.1 (0.0–0.1)	0.1 (0.0–0.

Certain conditions originating in the perinatal period are not relevant for cohorts 2 and 3.

*CI* confidence interval

International Classification of Diseases, Tenth Revision (ICD-10) codes – Infectious (ICD-10: A00-B99), Neoplasm (ICD-10: C00-D48), Blood (ICD-10: D50-D89), Endocrine (ICD-10: E00-E99), Mental (ICD-10: F00-F99), Nervous system (ICD-10: G00-G99), Eye (ICD-10: H00-H59), Ear (ICD-10: H60-H99), Circulatory (ICD-10: I00-I99), Respiratory (ICD-10: J00-J99), Digestive (ICD-10: K00-K93), Skin (ICD-10: L00-L99), Musculoskeletal (ICD-10: M00-M99), Genitourinary (ICD-10: N00-N99), Perinatal (ICD-10: P00-P96): Not relevant for cohort 2 and 3, Malformations (ICD-10: Q00-Q99), Other symptoms (ICD-10: R00-R99) and Injury (ICD-10: S00-T98)

Across all three cohorts and in most disease categories, the proportion of children registered with a diagnosis was higher the more severe the parental mental health condition.

Respiratory diseases (ICD-10: J00-J99) were the most commonly defined paediatric somatic morbidity across all three cohorts following infectious and parasitic diseases (ICD-10: A00-B99).

The overall number and total proportion of children diagnosed in each cohort and disease category is available in Supplementary table s2.

**Fig. 3:**
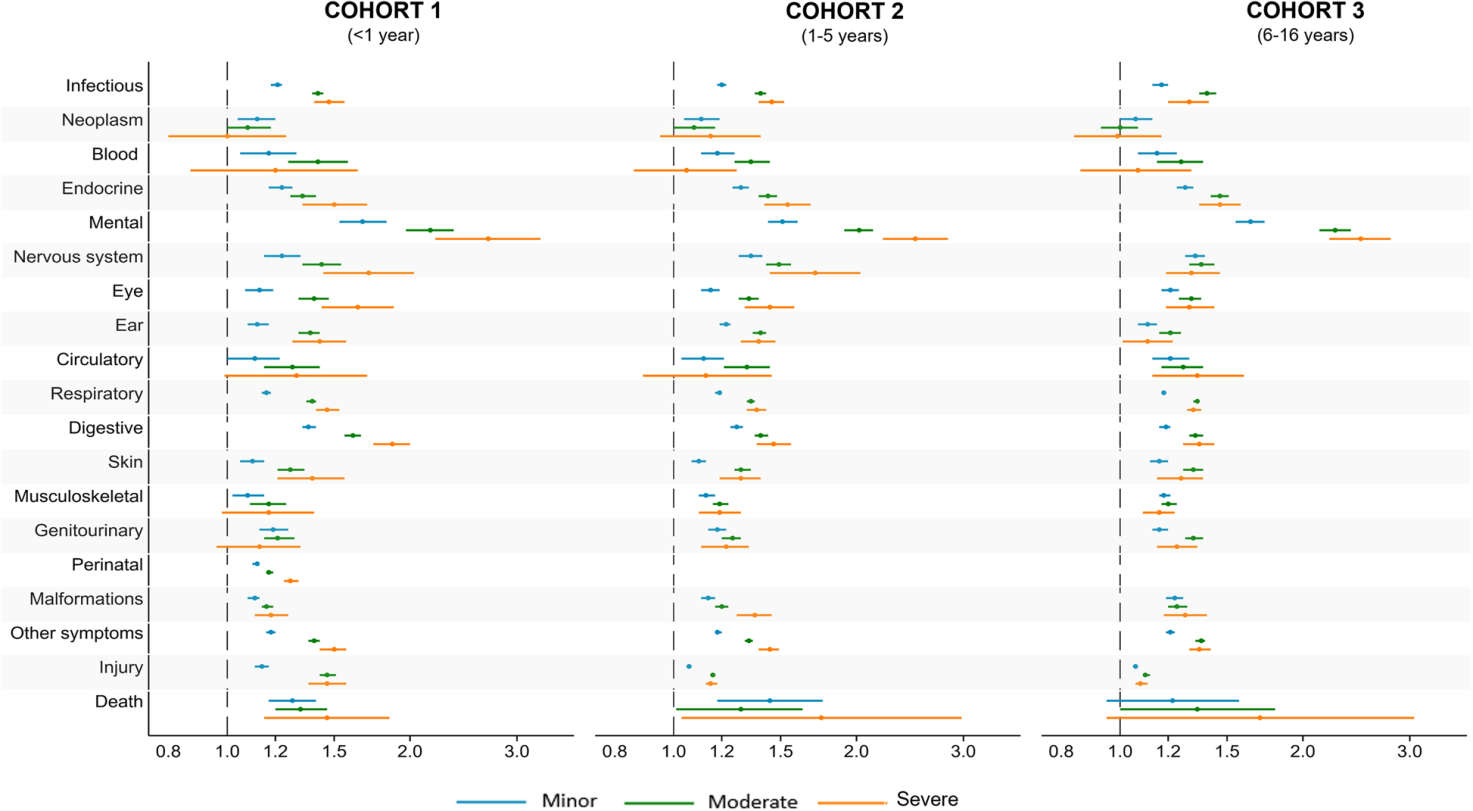
Risk ratio of registered diagnosis for children according to parental mental health conditions. **Legend:** Risk ratios for children exposed to different severities of parental mental health conditions with a registered diagnosis in each disease category were adjusted for birth year. International Classification of Diseases, Tenth Revision (ICD-10) codes** –** Infectious (ICD-10: A00-B99), Neoplasm (ICD-10: C00-D48), Blood (ICD-10: D50-D89), Endocrine (ICD-10: E00-E99), Mental (ICD-10: F00-F99), Nervous system (ICD-10: G00-G99), Eye (ICD-10: H00-H59), Ear (ICD-10: H60-H99), Circulatory (ICD-10: I00-I99), Respiratory (ICD-10: J00-J99), Digestive (ICD-10: K00-K93), Skin (ICD-10: L00-L99), Musculoskeletal (ICD-10: M00-M99), Genitourinary (ICD-10: N00-N99), Perinatal (ICD-10: P00-P96): Not relevant for cohort 2 and 3, Malformations (ICD-10: Q00-Q99), Other symptoms (ICD-10: R00-R99) and Injury (ICD-10: S00-T98)

Figure 3 presents the risk ratios (RR) of children with different severities of parental mental health conditions in every disease category within each cohort, compared to children with no parental mental health condition. The exact RR estimates and confidence intervals are available in Supplementary table s3. Besides mental and behavioural disorders in all three cohorts, the strongest association in cohort 1 was observed for diseases of the digestive system (ICD-10: K00-K99) where children with severe parental mental health conditions had 87% (95% CI: 74–100%) higher risk compared to children with no parental mental health conditions. In cohort 2 the strongest association was found for diseases within the nervous system (ICD-10: G00-G99) where children with severe parental mental health conditions had 83% (95% CI: 63–104%) higher risk. Not considering death, the strongest association in cohort 3 was observed for endocrine, nutritional and metabolic diseases (ICD-10: E00-E90) where children exposed to moderate parental mental health conditions had 46% (95% CI: 41–51%) and children with severe parental mental health conditions also had 46% (95% CI: 35–58%) higher risk than children not exposed.

Regarding death in all three cohorts, children exposed to parental mental health conditions had a higher risk. In cohort 1 children exposed to severe parental mental health conditions had a 46% (95% CI: 15–85%) higher risk of death than children not exposed. Similarly higher risk was found in children exposed in cohorts 2 and 3. However, in these two cohorts, only a few died during the study period resulting in very broad confidence intervals.

Overall, the analyses revealed a higher risk of morbidity in exposed children across all disease categories within each cohort except neoplasm in cohort 3. Furthermore, in most disease categories the overall results indicate a pattern of an increased risk of somatic morbidity in children the more severe the parental mental health condition (Fig. 3).

**Fig. 4:**
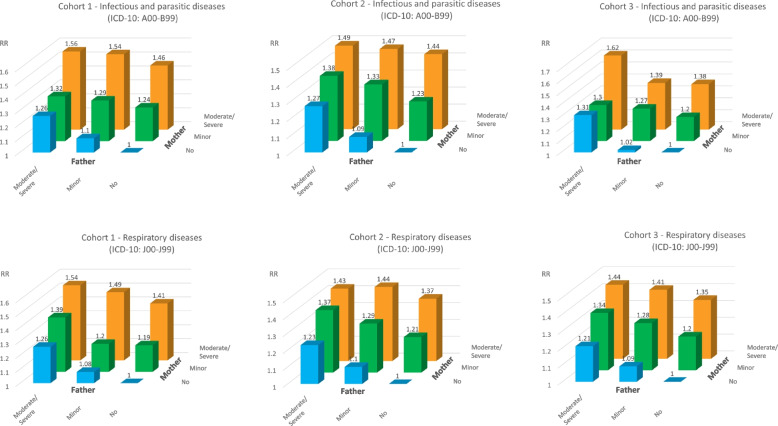
Risk ratio for infectious and respiratory diseases according to combined severity of paternal and maternal mental health conditions. Legend: Risk ratios were adjusted for birth year

Generally, RR of both respiratory and infectious diseases (the two most commonly defined diagnoses) were higher for children with only maternal mental health conditions, than for children exposed to only paternal mental health conditions (Fig. 4). However, children with paternal mental health conditions also had a higher risk than children with no parental mental health conditions. The risk was higher if both parents had a mental health condition, and children exposed to both a mother and father with moderate/severe mental health conditions had the highest risk of respiratory and infectious diseases. The confidence intervals are available in Supplementary table s4-s9.

## Discussion

In this nationwide cohort study, we describe and quantify a higher risk of somatic morbidity in children of different age-groups exposed to parental mental health conditions. Overall, this analysis discloses a somewhat poor physical health among these children indicated by an elevated risk across nearly all broad disease categories. Furthermore, in most disease categories and all age-groups of children, our results revealed a pattern of a higher risk the more severe the parental mental health condition. However, the large group of children exposed to minor parental mental health conditions also had a higher risk of somatic morbidity compared to children not exposed.

Both paternal and maternal mental health conditions were associated with a higher risk of infectious and respiratory diseases. However, associations were stronger for maternal mental health conditions than paternal. Children doubly exposed to a mother and father with moderate/severe mental health conditions had the most elevated risk of being diagnosed with an infectious and/or respiratory disease.

The direction of a higher risk of somatic morbidity across a broad range of disease categories in children with severe parental mental health conditions found in this explorative study is consistent with two other Danish nationwide studies [24, 25]. Although these children generally had the highest risk of being diagnosed, they merely concern a small proportion of the total study population. Our study revealed that children with minor parental mental health conditions also had a higher risk of poor physical health than children with no parental mental health conditions. This supports previous studies examining children exposed to minor parental mental health conditions that found a higher risk of asthma [34] and functional gastrointestinal disorder [35]. Thus, children with minor mental health conditions comprise a markedly large group that should not be disregarded.

It seems highly tempting to conclude that the higher risk of being diagnosed with somatic morbidity among children exposed to parental mental health conditions may be explained by an inappropriate parental utilization of healthcare. As reported by other studies, children of parents with mental health conditions are less likely to attend preventive healthcare such as routine childcare visits and childhood vaccinations [36, 37], though, more likely to use both primary and acute healthcare [38, 39]. Some might suggest that this higher frequency of healthcare use could be related to parental health anxiety. However, this study found that children with parental mental health conditions have a higher risk of being diagnosed with any somatic morbidity by a clinical specialist in secondary care. This may insinuate that the higher frequency of parental utilization of healthcare instead is compelled by a genuinely elevated need for healthcare regarding children with parental mental health conditions.

Mechanisms underlying this inequality are unknown, however, some evidence links early life and foetal life stressors, such as parental mental health conditions, to higher rates of mortality and morbidity from several chronic diseases through immune dysregulation [12]. Furthermore, children born of mothers with mental health conditions have a higher risk of being born prematurely [40] which affects both morbidity and mortality [41]. Although neonatal mortality has reduced [41], the association between maternal mental health conditions and preterm birth may especially explain some of the higher risk of death found in children aged below 1 year in cohort 1.

Other mechanisms could be related to parental health behaviour and health literacy, where parents’ mental health conditions may interfere with their capacity to understand and follow health advice from healthcare professionals on behalf of their child. Additionally, mental health conditions are associated with smoking and second-hand smoking in children has been causally linked to a higher risk of several diseases such as e.g. asthma and middle ear infections [42].

Mental health conditions may also negatively impact upon parenting behaviour [13–16], which could be a component in the poor physical health found in children with parental mental health conditions, as fundamental needs might be neglected. Our results indicate that a child with maternal mental health conditions and no paternal mental health conditions has a higher risk of infectious and/or respiratory diseases than a child with paternal mental health conditions and no maternal mental health conditions. This result might be explained by different gender household roles, where mothers most often take primary charge of child upbringing and overall care [43] and children more frequently cohabitate with their mother if parents do not cohabitate [44]. However, children with paternal mental health conditions still have a higher risk of somatic morbidity, thus, fathers and these children should not be neglected.

### Strengths and limitations

This study comprises several strengths by using high-quality and valid data from the national Danish registers covering the entire population [30, 45]. This enabled the examination of somatic morbidity in children exposed to different severities of parental mental health conditions across a broad range of disease categories in three large cohorts.

By including data on medication and data from primary care, we were able to include minor parental mental health conditions, which are often undiagnosed and treated alone by general practitioners [46], therefore often not included in register-based studies using hospital data [47]. To our knowledge, this is the only study including different severities of parental mental health conditions on child physical health.

There are also some limitations to this study. Obtaining information from the registers solely allowed for the identification of children exposed to parental mental health conditions if their parents actually had sought medical care. Therefore, some children whose parents had mental health conditions may have been misclassified as not exposed. This misclassification may have underestimated the association.

For some chronic diseases, recurrent children may occur in the three different cohorts, which may explain some of the similar patterns found in the three cohorts. However, this pattern is also found in e.g., infectious diseases which are not chronic. In addition, recurrent children thus are repeatedly hospitalized and diagnosed by a clinical specialist during the altered follow-up period within the different cohorts.

We did not consider if the child in fact was living with the parent suffering from mental health conditions, although severe parental mental health conditions, especially parental schizophrenia, are a strong predictor of the child being placed in out-of-home care [48]. However, children might still be affected by their mentally ill parent suffering although they are not living with that particular parent.

### Implication of findings

The overall higher risk of somatic morbidity predicted in children with minor, moderate or severe parental mental health conditions found in this study suggest a renewed emphasis upon these children’s physical health from both health professionals and politicians. Although, for some of the disease categories, the absolute differences were modest, we still find our results of great public health importance since parental mental health conditions concern a significantly large group of children [1]. Due to the excess cost used annually on e.g., hospital admissions among these children [9], targeted interventions may create public health benefits not only for the child and family, however also for government resources that are limited.

Our findings indicate that even within the first year of life children with parental mental health conditions require additional healthcare compared to children with no parental mental health conditions. This emphasises the need for early interventions perhaps even during pregnancy. From a public health perspective, it seems highly obvious that preventive interventions should be prioritised in these families. Such interventions may include supportive social networks and improvement of parenting behaviour and parental health literacy. However, solely based on this study no comprehensive improvement recommendations for such interventions can be made. Hence, future research is highly needed to examine other variables that yield the higher risk of somatic morbidity in children with different severities of parental mental health conditions observed in this study, in order to generate new hypotheses and hereafter concentrate on improving intervention strategies supporting these families.

## Conclusion

Children with parental mental health conditions experience an overall poor physical health with an elevated risk across most broad disease categories. Generally, the risk of somatic morbidity increased the more severe the parental mental health condition. However, minor parental mental health conditions should not be neglected since this concerns a substantially large group of children.

Children with both parents having moderate/severe mental health conditions were the most vulnerable to somatic morbidity. Furthermore, paternal, and maternal mental health conditions were individually associated with a higher risk of somatic morbidity. However, associations were stronger for maternal mental health conditions than paternal.

More support and awareness of children exposed to different severities of parental mental health conditions physical health is highly needed.

## Supplementary Information

**Additional file 1:** **Supplementary table s1.** Titles of overall disease category with corresponding ICD-10 codes and short description applied in Figs. 3 and 4 in main manuscript. **Supplementary table s2.** The overall number and total proportion of children diagnosed in each cohort and disease category. **Supplementary table s3.** Risk ratio (RR) and 95% confidence interval (CI) for children exposed to different severities of parental mental health conditions with a registered diagnoses in each disease category within all three cohorts. **Supplementary table s4.** Risk ratio for infectious diseases according to combined severity of paternal and maternal mental health conditions cohort 1. **Supplementary table s5.** Risk ratio for respiratory diseases according to combined severity of paternal and maternal mental health conditions cohort 1. **Supplementary table s6.** Risk ratio for infectious diseases according to combined severity of paternal and maternal mental health conditions cohort 2. **Supplementary table s7.** Risk ratio for respiratory diseases according to combined severity of paternal and maternal mental health conditions cohort 2. **Supplementary table s8.** Risk ratio for infectious diseases according to combined severity of paternal and maternal mental health conditions cohort 3. **Supplementary table s9.** Risk ratio for respiratory diseases according to combined severity of paternal and maternal mental health conditions cohort 3.

## Data Availability

The data that supports the results of this study is accessible from Statistics. Denmark. However, restrictions do apply to the availability, so data are not publicly available. Questions or requests regarding this data must be directed to the corresponding author Camilla Klinge Renneberg.
