# Supplementary material for: Children of parents with different severities of mental health conditions have higher risk of somatic morbidity: a Danish nationwide register-based cohort study
Source: BMC Public Health. 2023 May 3;23:810. doi: 10.1186/s12889-023-15714-8 (PMC10155386; doi:10.1186/s12889-023-15714-8)
Supplement: Supplementary file 1 — Additional file 1: Supplementary table s1. Titles of overall disease category with corresponding ICD-10 codes and short description applied in Figs. 3 and 4 in main manuscript. Supplementary table s2. The overall number and total proportion of children diagnosed in each cohort and disease category. Supplementary table s3. Risk ratio (RR) and 95% confidence interval (CI) for children exposed to different severities of parental mental health conditions with a registered diagnoses in each disease category within all three cohorts. Supplementary table s4. Risk ratio for infectious diseases according to combined severity of paternal and maternal mental health conditions cohort 1. Supplementary table s5. Risk ratio for respiratory diseases according to combined severity of paternal and maternal mental health conditions cohort 1. Supplementary table s6. Risk ratio for infectious diseases according to combined severity of paternal and maternal mental health conditions cohort 2. Supplementary table s7. Risk ratio for respiratory diseases according to combined severity of paternal and maternal mental health conditions cohort 2. Supplementary table s8. Risk ratio for infectious diseases according to combined severity of paternal and maternal mental health conditions cohort 3. Supplementary table s9. Risk ratio for respiratory diseases according to combined severity of paternal and maternal mental health conditions cohort 3. [file 12889_2023_15714_MOESM1_ESM.pdf]

BMC Public Health

Children of parents with different severities of mental health conditions have higher risk of somatic morbidity: A Danish nationwide register-based cohort study.

- Additional file 1 - Supplementary tables

Camilla Klinge Renneberg*[1]

René Børge Korsgaard Brund[1]

Signe Heuckendorff[1,2]

Bodil Hammer Bech[3]

Kirsten Fonager[1,4]

1. Department of Social Medicine, Aalborg University Hospital, Aalborg, Denmark
2. Psychiatry Region North Jutland, Aalborg, Denmark
3. Department of Public Health, Aarhus University, Aarhus, Denmark
4. Department of Clinical Medicine, Aalborg University, Aalborg, Denmark

*Corresponding author: Camilla Klinge Renneberg, ca-klinge@live.dk

Supplementary table of contents:

Supplementary table s1: Titles of overall disease category with corresponding ICD-10 codes and short description applied in Figure 3 and 4 in main manuscript

Supplementary table s2: The overall number and total proportion of children diagnosed in each cohort and disease category

Supplementary table s3: Risk ratio (RR) and 95% confidence interval (CI) for children exposed to different severities of parental mental health conditions with a registered diagnoses in each disease category within all three cohorts

Supplementary tables s4-s5: Risk ratio for infectious and respiratory diseases according to combined severity of paternal and maternal mental health conditions cohort 1

Supplementary tables s6-s7: Risk ratio for infectious and respiratory diseases according to combined severity of paternal and maternal mental health conditions cohort 2

Supplementary tables s8-s9: Risk ratio for infectious and respiratory diseases according to combined severity of paternal and maternal mental health conditions cohort 3

Supplementary

Supplementary table s1

Supplementary table s1 - Titles of overall disease category with corresponding ICD-10 codes and short description applied in Figure 3 and 4 in main manuscript

International Classification of Diseases, Tenth Revision (ICD-10)	ICD-10 codes	Short description in Figure 3 and 4
Certain infectious and parasitic diseases	A00-B99	Infectious
Neoplasms	C00-D48	Neoplasms
Diseases of the blood and blood-forming organs and certain disorders involving the immune mechanism	D50-D89	Blood
Endocrine, nutritional and metabolic diseases	E00-E90	Endocrine
Mental and behavioural disorders	F00-F99	Mental
Diseases of the nervous system	G00-G99	Nervous system
Diseases of the eye and adnexa	H00-H59	Eye
Diseases of the ear and mastoid process	H60-H95	Ear
Diseases of the circulatory system	I00-I99	Circulatory
Diseases of the respiratory system	J00-J99	Respiratory
Diseases of the digestive system	K00-K93	Digestive
Diseases of the skin and subcutaneous tissue	L00-L99	Skin
Diseases of the musculoskeletal system and connective tissue	M00-M99	Musculoskeletal
Diseases of the genitourinary system	N00-N99	Genitourinary
Certain conditions originating in the perinatal period	P00-P96	Perinatal
Congenital malformations, deformations and chromosomal abnormalities	Q00-Q99	Malformations
Symptoms, signs and abnormal clinical and laboratory findings, not elsewhere classified	R00-R99	Other symptoms
Injury, poisoning and certain other consequences of external causes	S00-T98	Injury

Supplementary table s2

Supplementary table s2 - The overall number and total proportion of children diagnosed in each cohort and disease category

**Disease grouping according to the ICD-10 classification and death**	**COHORT**					
	COHORT 1<br><1 year age group<br>(N= 1.028.587)		COHORT 2<br>1–5 years age group<br>(N= 965.253)		COHORT 3<br>6–16 years age group<br>(N= 680.799)	
	Total<br>**% (95% CI)**	**n**	Total<br>**% (95% CI)**	**n**	Total<br>**% (95% CI)**	**n**
**Infectious and parasitic diseases**	6.3 (6.2-6.3)	64,649	10.3 (10.2-10.3)	99,173	3.9 (3.8-3.9)	26,283
**Neoplasms**	0.6 (0.5-0.6)	5,680	0.7 (0.7-0.7)	7,026	1.0 (1.0-1.1)	6,991
**Diseases of the blood and blood-forming organs and certain disorders involving the immune mechanism**	0.3 (0.2-0.3)	2,576	0.8 (0.8-0.8)	7,671	0.6 (0.6-0.6)	4,208
**Endocrine, nutritional and metabolic diseases**	1.3 (1.2-1.3)	12,869	2.7 (2.6-2.7)	25,761	3.3 (3.2-3.3)	22,399
**Mental and behavioural disorders**	0.3 (0.3-0.3)	3,402	1.0 (1.0-1.0)	9,387	1.1 (1.1-1.1)	7,467
**Diseases of the nervous system**	0.6 (0.6-0.6)	6,289	1.5 (1.5-1.5)	14,371	2.3 (2.2-2.3)	15,449
**Diseases of the eye and adnexa**	0.9 (0.9-1.0)	9,616	2.5 (2.5-2.6)	24,551	2.6 (2.6-2.7)	17,982
**Diseases of the ear and mastoid process**	2.0 (1.9-2.0)	20,264	5.6 (5.6-5.7)	54,507	2.7 (2.7-2.7)	18,361
**Diseases of the circulatory system**	0.3 (0.3-0.3)	3,202	0.5 (0.5-0.5)	4,517	0.7 (0.7-0.8)	4,967
**Diseases of the respiratory system**	9.2 (9.2-9.3)	94,750	17.9 (17.8-18.0)	172,624	26.9 (26.8-27.0)	183,161
**Diseases of the digestive system**	3.2 (3.2-3.3)	33,088	5.2 (5.1-5.2)	49,922	6.1 (6.0-6.1)	41,426
**Diseases of the skin and subcutaneous tissue**	1.3 (1.3-1.4)	13,884	3.7 (3.7-3.8)	36,179	3.1 (3.1-3.2)	21,342
**Diseases of the musculoskeletal system and connective tissue**	0.8 (0.8-0.8)	8,321	3.8 (3.8-3.9)	37,002	7.1 (7.0-7.1)	48,226
**Diseases of the genitourinary system**	1.0 (1.0-1.1)	10,725	3.1 (3.0-3.1)	29,456	3.9 (3.9-4.0)	26,778
**Certain condition originating in the perinatal period**	27.2 (27.1-27.3)	279,412	-		-	
**Congenital malformations and chromosomal abnormalities**	5.9 (5.9-6.0)	61,016	5.1 (5.0-5.1)	48,961	3.6 (3.6-3.7)	24,568
**Symptoms, signs and abnormal clinical and laboratory findings, not elsewhere classified**	8.2 (8.1-8.2)	84,194	14.5 (14.4-14.5)	139,555	13.9 (13.9-14.0)	94,856
**Injury, poisoning and certain consequences of external causes**	4.0 (4.0-4.1)	41,542	41.7 (41.6-41.8)	402,574	48.0 (47.9-48.1)	326,926
**Death**	0.4 (0.4-0.4)	3,951	0.1 (0.1-0.1)	669	0.1 (0.0-0.1)	365

Supplementary table s3

Supplementary table s3: Risk ratio (RR) and 95% confidence interval (CI) for children exposed to different severities of parental mental health conditions with a registered diagnoses in each disease category within all three cohorts

Disease grouping according to the ICD-10 classification and mortality/death	COHORT								
	COHORT 1 <1 year age group (N=1.028.587)			COHORT 2 1–5 years age group (N= 965.253)			COHORT 3 6–16 years age group (N= 680.799)		
	MENTAL HEALTH CONDTION								
	Minor	Moderate	Severe	Minor	Moderate	Severe	Minor	Moderate	Severe
	RR (95% CI)	RR (95% CI)	RR (95% CI)	RR (95% CI)	RR (95% CI)	RR (95% CI)	RR (95% CI)	RR (95% CI)	RR (95% CI)
Infectious and parasitic diseases	1.21 (1.18-1.23)	1.41 (1.38-1.44)	1.47 (1.39-1.56)	1.20 (1.18-1.22)	1.39 (1.36-1.42)	1.45 (1.38-1.52)	1.17 (1.13-1.20)	1.39 (1.35-1.44)	1.30 (1.20-1.40)
Neoplasms	1.12 (1.04-1.20)	1.08 (1.00-1.18)	1.00 (0.80-1.25)	1.11 (1.04-1.19)	1.08 (1.00-1.17)	1.15 (0.95-1.39)	1.06 (1.00-1.13)	1.00 (0.93-1.07)	0.99 (0.84-1.17)
Diseases of the blood and blood-forming organs and certain disorders involving the immune mechanism	1.17 (1.05-1.30)	1.41 (1.26-1.58)	1.20 (0.87-1.64)	1.18 (1.11-1.26)	1.34 (1.26-1.44)	1.05 (0.86-1.27)	1.15 (1.07-1.24)	1.26 (1.15-1.37)	1.07 (0.86-1.31)
Endocrine, nutritional and metabolic diseases	1.23 (1.17-1.28)	1.33 (1.27-1.40)	1.50 (1.33-1.70)	1.29 (1.25-1.33)	1.43 (1.38-1.48)	1.54 (1.41-1.68)	1.28 (1.24-1.32)	1.46 (1.41-1.51)	1.46 (1.35-1.58)
Mental and behavioural disorders	1.67 (1.53-1.83)	2.16 (1.97-2.36)	2.69 (2.20-3.28)	1.51 (1.43-1.60)	2.02 (1.91-2.13)	2.50 (2.21-2.83)	1.64 (1.55-1.73)	2.26 (2.13-2.40)	2.49 (2.21-2.79)
Diseases of the nervous system	1.23 (1.15-1.32)	1.43 (1.33-1.54)	1.71 (1.44-2.03)	1.34 (1.28-1.40)	1.49 (1.42-1.56)	1.83 (1.63-2.04)	1.33 (1.28-1.38)	1.36 (1.30-1.43)	1.31 (1.19-1.46)
Diseases of the eye and adnexa	1.13 (1.07-1.19)	1.39 (1.31-1.47)	1.64 (1.43-1.88)	1.15 (1.11-1.19)	1.33 (1.28-1.38)	1.44 (1.31-1.58)	1.21 (1.17-1.25)	1.31 (1.25-1.36)	1.30 (1.19-1.43)
Diseases of the ear and mastoid process	1.12 (1.08-1.17)	1.37 (1.31-1.42)	1.42 (1.28-1.57)	1.22 (1.19-1.24)	1.39 (1.35-1.42)	1.38 (1.29-1.47)	1.11 (1.07-1.15)	1.21 (1.16-1.26)	1.11 (1.01-1.22)
Diseases of the circulatory system	1.11 (1.00-1.22)	1.28 (1.15-1.42)	1.30 (0.99-1.70)	1.12 (1.03-1.21)	1.32 (1.21-1.44)	1.13 (0.89-1.45)	1.21 (1.13-1.30)	1.27 (1.17-1.37)	1.34 (1.13-1.60)
Diseases of the respiratory system	1.16 (1.14-1.18)	1.38 (1.35-1.40)	1.46 (1.40-1.53)	1.19 (1.17-1.20)	1.34 (1.32-1.36)	1.37 (1.32-1.42)	1.18 (1.17-1.19)	1.34 (1.32-1.35)	1.32 (1.29-1.36)
Diseases of the digestive system	1.36 (1.33-1.40)	1.61 (1.56-1.66)	1.87 (1.74-2.00)	1.27 (1.24-1.30)	1.39 (1.36-1.43)	1.46 (1.37-1.56)	1.19 (1.16-1.21)	1.33 (1.30-1.37)	1.35 (1.27-1.43)
Diseases of the skin and subcutaneous tissue	1.10 (1.05-1.15)	1.27 (1.21-1.34)	1.38 (1.21-1.56)	1.10 (1.07-1.13)	1.29 (1.26-1.34)	1.29 (1.19-1.39)	1.16 (1.12-1.20)	1.32 (1.27-1.37)	1.26 (1.15-1.37)
Diseases of the musculoskeletal system and connective tissue	1.08 (1.02-1.15)	1.17 (1.09-1.25)	1.17 (0.98-1.39)	1.13 (1.10-1.17)	1.19 (1.16-1.23)	1.19 (1.10-1.29)	1.18 (1.16-1.21)	1.20 (1.17-1.24)	1.16 (1.09-1.23)
Diseases of the genitourinary system	1.19 (1.13-1.26)	1.21 (1.15-1.29)	1.13 (0.96-1.32)	1.18 (1.14-1.22)	1.25 (1.20-1.29)	1.22 (1.11-1.33)	1.16 (1.13-1.20)	1.32 (1.28-1.37)	1.24 (1.15-1.34)
Certain condition originating in the perinatal period	1.12 (1.10-1.13)	1.17 (1.16-1.19)	1.27 (1.24-1.31)	-	-	-	-	-	-
Congenital malformations and chromosomal abnormalities	1.11 (1.08-1.13)	1.16 (1.14-1.19)	1.18 (1.11-1.26)	1.14 (1.11-1.17)	1.20 (1.17-1.23)	1.36 (1.27-1.45)	1.23 (1.19-1.27)	1.24 (1.20-1.29)	1.28 (1.18-1.39)
Symptoms, signs and abnormal clinical and laboratory findings. not elsewhere classified	1.18 (1.16-1.20)	1.39 (1.36-1.42)	1.50 (1.42-1.57)	1.18 (1.17-1.20)	1.33 (1.31-1.35)	1.44 (1.38-1.49)	1.21 (1.19-1.23)	1.36 (1.33-1.38)	1.35 (1.30-1.41)
Injury, poisoning and certain consequences of external causes	1.14 (1.11-1.17)	1.46 (1.42-1.51)	1.46 (1.36-1.57)	1.06 (1.05-1.07)	1.16 (1.15-1.17)	1.15 (1.13-1.18)	1.06 (1.05-1.07)	1.10 (1.09-1.12)	1.08 (1.06-1.11)
Death	1.28 (1.17-1.40)	1.32 (1.20-1.46)	1.46 (1.15-1.85)	1.44 (1.18-1.76)	1.29 (1.01-1.63)	1.75 (1.03-2.98)	1.22 (0.95-1.57)	1.34 (1.00-1.80)	1.70 (0.95-3.05)

Supplementary table s4

Supplementary table s4: Risk ratio for infectious diseases according to combined severity of paternal and maternal mental health conditions cohort 1

Cohort 1<br><br>Infectious diseases (ICD-10: A00-B99)	Father			
	MENTAL HEALTH CONDITION	No	Minor	Moderate/Severe
Mother	No	1 (ref)	1.10 (1.06-1.14)	1.26 (1.21-1.31)
	Minor	1.24 (1.21-1.27)	1.29 (1.21-1.36)	1.32 (1.23-1.43)
	Moderate/Severe	1.46 (1.42-1.50)	1.54 (1.44-1.65)	1.56 (1.47-1.66)

Supplementary table s5

Supplementary table s5: Risk ratio for respiratory diseases according to combined severity of paternal and maternal mental health conditions cohort 1

Cohort 1<br><br>Respiratory diseases (ICD-10: J00-J99)	Father			
	MENTAL HEALTH CONDITION	No	Minor	Moderate/Severe
Mother	No	1 (ref)	1.08 (1.04-1.11)	1.26 (1.22-1.31)
	Minor	1.19 (1.16-1.21)	1.20 (1.14-1.26)	1.39 (1.30-1.48)
	Moderate/Severe	1.41 (1.37-1.44)	1.49 (1.40-1.58)	1.54 (1.47-1.62)

Supplementary table s6

Supplementary table s6: Risk ratio for infectious diseases according to combined severity of paternal and maternal mental health conditions cohort 2

**Cohort 2**<br><br>**Infectious diseases (ICD-10: A00-B99)**	**Father**			
**Mother**	**MENTAL HEALTH CONDITION**	**No**	**Minor**	**Moderate/Severe**
	**No**	1 (ref)	1.09 (1.05-1.12)	1.27 (1.23-1.31)
	**Minor**	1.23 (1.20-1.25)	1.33 (1.28-1.39)	1.38 (1.30-1.47)
	**Moderate/Severe**	1.44 (1.40-1.47)	1.47 (1.39-1.55)	1.49 (1.42-1.57)

Supplementary table s7

Supplementary table s7: Risk ratio for respiratory diseases according to combined severity of paternal and maternal mental health conditions cohort 2

**Cohort 2**<br><br>**Respiratory diseases (ICD-10: J00-J99)**	**Father**			
**Mother**	**MENTAL HEALTH CONDITION**	**No**	**Minor**	**Moderate/Severe**
	**No**	1 (ref)	1.10 (1.07-1.13)	1.23 (1.19-1.26)
	**Minor**	1.21 (1.20-1.23)	1.29 (1.24-1.33)	1.37 (1.31-1.44)
	**Moderate/Severe**	1.37 (1.34-1.39)	1.44 (1.38-1.50)	1.43 (1.38-1.49)

Supplementary table s8

Supplementary table s8: Risk ratio for infectious diseases according to combined severity of paternal and maternal mental health conditions cohort 3

**Cohort 3**<br><br>**Infectious diseases (ICD-10: A00-B99)**	**Father**			
**Mother**	**MENTAL HEALTH CONDITION**	**No**	**Minor**	**Moderate/Severe**
	**No**	1 (ref)	1.02 (0.97-1.08)	1.31 (1.23-1.39)
	**Minor**	1.20 (1.16-1.24)	1.27 (1.19-1.36)	1.30 (1.19-1.41)
	**Moderate/Severe**	1.38 (1.32-1.44)	1.39 (1.27-1.52)	1.62 (1.49-1.75)

Supplementary table s9

Supplementary table s9: Risk ratio for respiratory diseases according to combined severity of paternal and maternal mental health conditions cohort 3

**Cohort 3**<br><br>**Respiratory diseases (ICD-10: J00-J99)**	**Father**			
**Mother**	**MENTAL HEALTH CONDITION**	**No**	**Minor**	**Moderate/Severe**
	**No**	1 (ref)	1.09 (1.07-1.11)	1.21 (1.18-1.24)
	**Minor**	1.20 (1.18-1.21)	1.28 (1.24-1.31)	1.34 (1.30-1.39)
	**Moderate/Severe**	1.35 (1.33-1.37)	1.41 (1.36-1.46)	1.44 (1.40-1.49)
